# Medicine

**Published:** 1910-06

**Authors:** J. M. Fortesque-Brickdale


### Progress of the Medical Sciences.

### MEDICINE.

**Epilepsy and its Treatment.**—Considerable attention has recently been given to this important subject, which now is accorded the honour of a journal solely devoted to its discussion, and appropriately named *Epilepsia*. A masterly account of the present position is given by Dr. W. Aldren Turner in his Morison Lectures, which were published in the *British Medical Journal* during March and April of the present year. As regards treatment, Dr. Turner points out that in all cases it is the patient and not the disease merely that requires treatment. All sources of peripheral irritation—*e.g.* adenoids, errors of refraction, dental caries, dyspepsia, etc.—should be removed, and a fair amount of exercise, hot baths and douches, and an out-of-door life are to be encouraged. Mental exercise of an ordinary kind should not be forbidden, unless the fits have been brought on by the strain of an examination or some similar cause. As regards drugs, "there is no single specific remedy in the treatment of epilepsy, although the alkaline salts of bromine come nearest to this definition. But the influence of the bromides on epileptic convulsions is variable and uncertain." Among 366 cases, in 23.5 per cent. the fits were arrested, in 28.7 per cent. there was improvement, and in 47.8 per cent. there was no improvement, or the frequency and severity of the attacks was actually increased. These figures are in accordance with those of other authorities. The question of dosage is important. Moderate doses (45 to 90 grains daily) of bromine salts are recommended; larger doses only set up bromism, and do not produce any permanent good: the drug should be taken for at least two years. Dr. Turner prefers the sodium salt, but notes that the strontium salt is also useful, and may be given in smaller doses. Dr. Taylor [1] practically coincides with Dr. Aldren Turner in his method of administration of bromides, at the same time remarking that the strontium salts are not liable to cause acne. In all cases he combines nux vomica with the bromides, which he says prevents the general depression consequent on a long course of bromide alone. Both these authorities agree also in recommending digitalis when the ordinary indications for that drug, especially low arterial pressure, are present.

A considerable amount of evidence has now accumulated as to the value of **calcium salts** in epilepsy, although it is not clear yet what class of case is likely to be benefited. Donath, [2] in *Epilepsia*, has summarised recent investigations on this subject, and also recorded nine cases of his own, in which the results were not, however, satisfactory. The use of these salts was based on Sabbatani's observations to the effect that calcium salts lowered the excitability of the cortical cells, whereas oxalates and citrates produce an opposite effect. It was then thought that possibly some connection between the coagulation time of the blood and the frequency of the fits might be established, but the results of Besta's and Perugia's experiments, which showed a diminution of coagulability directly proportional to the number of fits, have not been confirmed by others. Equally discordant are the results of those who, like Stoltzner, Netter, Cohn and Quest, tried to prove a diminution in the calcium content of the brains of infants who had died of tetany, or lowered sensibility to galvanism during life.

Lallemant and Dupouy [3] gave 3 grammes (45 grains) calcium chloride per diem to fourteen adult women for six months, but obtained negative results in all cases.

Littlejohn, [4] on the other hand, giving 15 grains calcium lactate thrice daily was able to report very favourably on the action of the drug in otherwise obstinate cases.

Observations by Dr. Muskens, [5] also published in *Epilepsia*, point to a new method of treating the epileptic attack based upon the power to foresee its occurrence. He observed that previously to the fit certain sensory alterations occurred in the form of segmental analgesias or areas of diminished sensibility,

[1] *Lancet*, 1910, i, 354. [2] *Brit. M. J.*, 1910, i, 281.
[3] *Gaz. d. Hôp.*, 1910, lxxxiii, 712. [4] *Lancet*, 1909, i, 1,382.
[5] *Brit. M. J.*, 1910, i, 769.

which were replaced by hyperæsthetic areas after the discharge had taken place. The frequency with which this phenomenon was noted appears to vary somewhat. Muskens states that it occurs in 44 per cent. of his cases, but other observers have recorded it in as many as 80 per cent. Where this prodromal warning occurs, the author finds that he can often abort the fit by increasing the dose of bromide, and at the same time administering a sharp purgative.

**Nicotine and Tobacco Smoke.** Up till a few years ago, it was very generally believed that tobacco smoke owed most of its toxic properties to the so-called "pyridine bases"—bodies derived from an aromatic substance having the formula $C_5H_5N$, that is benzine in which one CH group has been replaced by nitrogen. The alkaloid nicotine, which is itself a pyridine derivative, was thought to be decomposed at the temperature of ignited tobacco. As was pointed out, however, by Dixon Mann [1] in a short paper on the effects of excessive smoking, there has been a considerable accumulation of recent evidence in favour of nicotine being the chief poisonous constituent of tobacco smoke. Not only have numerous observers shown that nicotine is present in considerable amounts in the smoke, but from the physiological side also experiments have been carried out proving that the symptoms of tobacco smoke poisoning in animals are identical with those produced by nicotine. On the other hand no effect was produced either in man or animals when tobacco freed from nicotine was employed experimentally, although in this form the "pyridine bases" were undoubtedly present. The fact that in many cases of arterio-sclerosis the original hypertension appears to have been due to excessive use of tobacco is to a certain extent born out by animal experiment, though the arterial changes are not altogether those of ordinary arterio-sclerosis, but tend (in rabbits) to produce rather atheroma of the aorta.

It is, however, certain that there are other important poisons besides nicotine in tobacco smoke, and one of the chief of these is carbon monoxide. CO-hæmoglobin may be produced experimentally by passing small amounts of smoke over a dilute solution of blood. [2] It is stated that one ounce of tobacco smoked in the form of cigarettes gives off from one to four pints of carbon monoxide, while the same amount in a pipe gives off from two and a quarter to five pints. As little as 0.15 per cent. CO in the atmosphere is usually considered dangerous, and 0.4 per cent. is fatal to animals. These observations certainly tend to show that, apart from other considerations, the practice of inhaling cigarette smoke is one of the factors which renders this form of

[1] *Ibid.*, 1908, ii, 1,673. [2] *Lancet*, 1908, ii, 104.

tobacco more generally toxic than the other two. Other constituents of tobacco smoke are stated by Dixon Mann to comprise ammonia, methylamine, hydrocyanic acid, sulphuretted hydrogen and tarry products (higher phenols). Recently[1] it has been shown that thiocyanates, probably of ammonium, may be added to this list without, however, any great increase in the physiological effect, as the complex ion SCN does not produce the typical cyanogen symptoms.

A curious case in which tobacco was ingested unburnt has been recorded by Lemaire.[2] An infant just over one year of age swallowed one of Wills's "Three Castles" cigarettes, containing rather more than 1 gramme (15 grains) of tobacco. This took place at 6 p.m., and there were no results for an hour or two, when diarrhœa, vomiting and collapse set in, both fæces and vomited matter containing portions of the cigarette. The pulse and respiration were not disturbed, and warm stimulants restored the child, who subsequently went to sleep and appeared to be none the worse by the following day.

Investigations carried out last year[3] showed that, speaking generally, the proportion of nicotine in a given tobacco, cigar or cigarette, corresponded with its strength or mildness from the smoker's point of view. Good quality Virginian cigarettes contained nearly twice as much nicotine as Egyptians, and common Virginians sold at five a penny, though showing a smaller amount than the better sort, still contained more than either Turkish or Egyptian brands. The latter observation may possibly be explained by the fact that cheap Virginian cigarettes are not solely composed of tobacco. A cigar, a pipe, and four Egyptian cigarettes, all took about the same time to smoke—namely 50 minutes—and the amount of nicotine contained in each would be 0.9 grain, 1.1 grain, and 0.9 grain respectively, though of course it is not thereby shown that the alkaloid is absorbed by the smoker in like ratios : indeed, it seems probable that the pipe is by far the least harmful method of smoking,[4] due regard being had to the "strength" of the tobacco employed. The condensation products mainly collect in the stem and bottom of the bowl, and the smoke is not so commonly inhaled or swallowed. In addition to this, as Dixon Mann pointed out in the paper to which reference has already been made, nicotine is absorbed through the unbroken skin, and the discoloured fingers of the habitual cigarette smoker must therefore be the means of entrance for some of the poison into his body. It is, however, probable that the complete access of air to the cigarette renders combustion more complete, though possibly this only means the formation of more carbon monoxide. The cigar is

[1] *Ibid.* [2] *Ann. de Méd. et de Chir.*, March, 1909.
[3] *Brit. M. J.*, 1909, i, 919., [4] *Lancet*, 1910, i, 314.

generally considered the most dangerous form in which tobacco can be taken, mainly owing to the fact that the products of condensation travel gradually into the "proximal" end, and so into the mouth of the smoker.

It will be very consoling for the habitual smoker to learn that there is some evidence to show that tobacco may give protection against epidemic cerebro-spinal meningitis.[1] Out of 43 soldiers in a French regiment suffering from this disease, of whom exact particulars could be obtained, it was found that 25.2 per cent. were smokers, 30.2 per cent. "occasional" smokers, and 44 per cent. non-smokers. The smokers in the regiment were estimated at 94 per cent. of the total strength, so that it was concluded that some protection was afforded by the habit. Moreover, as to virulence, among nine fatal cases only one was a smoker, four were "occasional," and the rest non-smokers. Apparently regular and persistent smoking is necessary to secure any degree of protection, and should further and more extensive observations support the conclusions derived from this preliminary study, many will no doubt feel considerable satisfaction in knowing that some really valuable result has been attained by their painstaking efforts to create "records" in their annual or daily consumption of the weed.

Skaller,[2] moreover, by making a solution of cigar and cigarette smoke, and injecting it into dogs with Pawlow's fistula, showed that the products of combustion caused a considerable increase in gastric secretion. A solution of .02 gm. pure nicotine had the same effect, whereas smoke from "nicotine-free" cigars gave a negative result. He concludes that after a heavy meal a cigar may actually improve digestion, though he notes that actual hypersecretion is an earlier symptom of nicotine poisoning than the well-known central scotomata.

J. M. FORTESCUE-BRICKDALE.

